# Fertility awareness and attitudes towards parenthood among Danish university college students

**DOI:** 10.1186/s12978-016-0258-1

**Published:** 2016-12-13

**Authors:** Nina Olsén Sørensen, Signe Marcussen, Mette Grønbæk Backhausen, Mette Juhl, Lone Schmidt, Tanja Tydén, Hanne Kristine Hegaard

**Affiliations:** 1 The Research Unit Women’s and Children’s Health, section 7821, The Juliane Marie Centre for Women, Children and Reproduction, Copenhagen University Hospital, Rigshospitalet, Blegdamsvej 9, 2100 Copenhagen, Denmark; 2 Department of Obstetrics, Copenhagen University Hospital, Rigshospitalet, Blegdamsvej 9, 2100 Copenhagen, Denmark; 3 Midwifery Program, Metropolitan University College, Sigurdsgade 26, 2200 Copenhagen, Denmark; 4 Department of Gynecology and Obstetrics, Zealand University Hospital, Syghusvej 10, 4000 Roskilde, Denmark; 5 Department of Public Health, University of Copenhagen, Øster Farimagsgade 5, 1014 Copenhagen, Denmark; 6 Department of Women’s and Children’s Health, Uppsala University, Akademiske Sjukhuset, SE-751 85 Uppsala, Sweden

**Keywords:** Postponed parenthood, Fertility awareness, Parenting attitudes, Questionnaire

## Abstract

**Background:**

Postponing parenthood has steadily increased during the past decades in Western countries. This trend has affected the size of families in the direction of fewer children born per couple. In addition, higher maternal age is associated with an increased risk of pregnancy-related complications such as prematurity and foetal death, while higher paternal age increases the risk of miscarriage and affects time-to-pregnancy. Hence, understanding the circumstances and reflections that influence the decision is greatly needed and little is known about potential gender difference influencing the choice. The aim was to investigate attitudes towards parenthood, intentions for childbirth and knowledge about fertility issues among men and women*.*

**Methods:**

We conducted a cross-sectional study based on a validated 49-item questionnaire among students, who attended selected mandatory lectures at a Danish university college in February to April 2016. The participation rate was 99%, and 517 completed the questionnaire.

**Results:**

Though the majority of all participants wished to have children in the future (>86%), there was significant difference between the genders (*p* = 0.002). Women rated having children to be more important than men did (*p* < 0.001), while men rated higher the likelihood of abstaining from having children if faced with infertility (*p* = 0.003). Knowledge about fertility issues was similar between genders including poor knowledge about the age-related decline in female fertility. While women found it more important to have children before being ‘too old’ (*p* = 0.04), still more than 40% of all respondents intended to have their last child after the age of 35 years. For both genders the most important prerequisite for parenthood was having a partner to share responsibility with. Perceived or experienced life changes related to parenthood were generally positive such as personal development.

**Conclusion:**

The majority of respondents wished to have children, but many desired to have these after the biological decline in female fertility. The moderate knowledge level among both genders uncovered in this study is of concern. Future research should address the potential link between fertility knowledge and planning of parenthood. We may benefit from intervention studies examining the effect of routine preconception care.

## Plain English Summary

During the past decades an increase in parental age has been seen in Western countries. This trend has affected the size of families in the direction of fewer children born per couple - in part due to the biological decline in fertility. Understanding what influences the decision to postpone pregnancy is greatly needed and little is known about potential gender difference influencing the choice. The aim was to investigate attitudes towards parenthood, intentions for childbirth and knowledge about fertility issues among both men and women*.*

We conducted a Danish questionnaire study in the period February to April 2016. The study population was 517 university college students and the participation rate was 99%. The majority of these wished to have children in the future, but there was a significant difference between the genders. Women stated having children as being more important, while men were more likely to abstain from having children if faced with infertility. Knowledge about fertility issues was similar among both genders including poor knowledge about the age-related decline in female fertility. More than 40% intended to have their last child after the age of 35 years. The most important prerequisite for parenthood was having a partner to share responsibility with. Perceived or experienced life changes were generally positive such as personal development.

Good reproductive health starts before pregnancy and entails reflection about family planning and knowledge about fertility. Future research should address the potential link between fertility knowledge and planning of parenthood.

## Background

During the past decades many countries have seen a marked increase in parental age [1, 2]. In Denmark the age of first time fathers and mothers has increased by three and four years, respectively, since 1986 - resulting in a mean age of 31.3 years for men and 29.1 years for women in 2015 [3]. Similar trends are seen in other countries [1], and across the European Union first time mothers are oldest in Italy (30.6 years) and Spain (30.4 years) [4]. In the USA the proportion of mothers aged 35 years or older has steadily increased over the last 25 years, but the mean age of first time mothers is lower than in Europe (26.0 years in 2013) [5].

Advanced age of the mother, as well as the father, is known to be related to reduced fertility [6, 7], and the delay of parenthood seems to affect family size in the direction of fewer children born per couple [8]. Accordingly, a decline in total fertility rate (TFR) has been seen in OECD countries, where the average TFR dropped from 2.7 to 1.7 during the years 1970 to 2009 [9]. Higher maternal age is also associated with an increase in pregnancy-related complications and adverse outcome in the offspring [7] such as prematurity [10] and foetal death [11, 12]. With regards to paternal age, studies have found an association with the general reproductive function such as prolonged time-to-pregnancy, and with pregnancy outcomes such as miscarriage [7]. Postponement of parenthood moreover implies that medically assisted reproduction (MAR) is a reality for a growing number of couples. However, the biological decline in fertility by advanced parental age cannot fully be compensated for by MAR [13], and consequently society as a whole is affected. Moreover, the psychological strain of undergoing fertility treatment should not be ignored [14]. A large Finnish register-based study found that infertile women who had received MAR-treatment, that did not result in a childbirth, had higher rates of hospitalizations for psychiatric diagnoses compared with MAR-treated women, who did give birth [15].

The present study is a survey among university college students in Denmark. The fertility patterns in the Nordic welfare countries is quite similar. A demographic study covering national data from Denmark, Finland, Norway and Sweden on cohort fertility among women born in 1935 and later have shown similar patterns in the four countries regarding postponement of family formation and a recuperation in fertility levels at ages 30 and above in younger cohorts [16]. At 40 years of age women born in 1935 in the Nordic countries achieved on average 2.1–2.5 children, and women born in 1963 achieved on average 1.9–2.1 children [16]. Postponement of family formation was seen across all educational groups, however, the postponement was more pronounced among highly educated women [16]. In Denmark, in 2005 37-year old men had on average 1.5 children, and there were no differences in average number of children across educational groups. Among 35-year old women short-term educated women had on average 1.9 children compared to long-term educated women having on average 1.4 children [17]. Based on Danish national register data, since 2005 12–13% of 50-year old women are childless compared to 20–21% among men [18]. Based on register data it is not possible to discriminate between voluntarily childlessness and childlessness due to infertility. In Denmark, around 86% of 30–39-year old fathers and 77% of mothers are at the labor market and 97% of all 3–5 years old children are attending public day child care [19]. Danish parents have together up to 52 weeks of parental leave, whereof 32 weeks can be shared between the parents. In total, 37% of fathers use full/some part of the shared parental leave [20].

From a public health perspective and in the light of the abovementioned adverse medical and psychological consequences, the understanding of underlying factors for parenthood postponement warrants research and attention. Previous studies have uncovered several reasons including contemporary norms, the rise in access to and effectiveness of contraception, and an increase in women’s level of education and labor market participation [21, 16]. General lack of fertility knowledge, including the age-related decline in fertility, may also be a central and contributing factor [21–23]. While preconception care – comprising of counseling about fertility *-* is not routinely provided in Denmark, recent campaigns run by the Danish National Health Authority [24] have addressed the issue of fertility awareness. However, the effect of these campaigns is not known. Another central issue is whether there are gender differences in fertility knowledge [22, 25].

## Aim

The aim was to investigate attitudes towards parenthood, intentions for childbirth, and knowledge about fertility issues among male and female university college students.

## Methods

We conducted a cross-sectional study in Copenhagen, Denmark, during a two-month period of February to April 2016. The Danish Data Protection Agency (j.nr: 2012-58-0004), as well as the administration at the Metropolitan University College, approved the study.

### Study population

The study was carried out among male and female students enrolled in a full degree study program at the Metropolitan University College, Copenhagen, where a range of professional bachelor degree programs are offered (see Table 1)*.* All of the 995 students registered to attend a mandatory module called the Inter-professional module were considered eligible. We chose to recruit from this module, because it was an ideal opportunity to engage students from all bachelor programmes and because of the recruitment convenience of large classroom or auditorium lectures.

### Procedure

We recruited the students in continuation of a lecture, and one of three authors (NOS, SM and MJ) gave a brief oral introduction to the purpose of the study using layman terms. Students were asked to fill out a 15-minute written questionnaire on attitudes towards parenthood, intentions for childbirth and knowledge about fertility issues. It was emphasized that participation was voluntary and anonymous, and that they could hand in a blank questionnaire. The use of aids, e.g. the internet, was discouraged. The questionnaires were handed out in envelopes and likewise collected immediately after being filled out. Among the 527 students present, 520 wanted to participate in the study leaving a participation rate of 99%. Blank questionnaires (*n* = 3) were excluded from the data analysis leaving a final study population of 517.

### Questionnaire and study variables

**Fig. 1:**
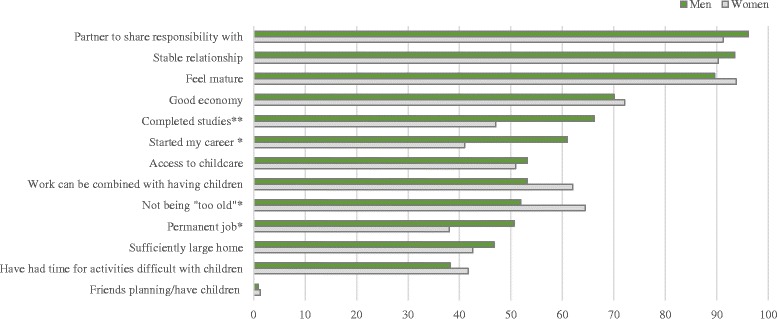
Circumstances of importance. ªTo illustrate the participants’ main responses the total percentage for responses ‘important’ or ‘very important’ are presented. Significant effect of having children among women on variables: *p < 0.05; **p < 0.001

**Fig. 2:**
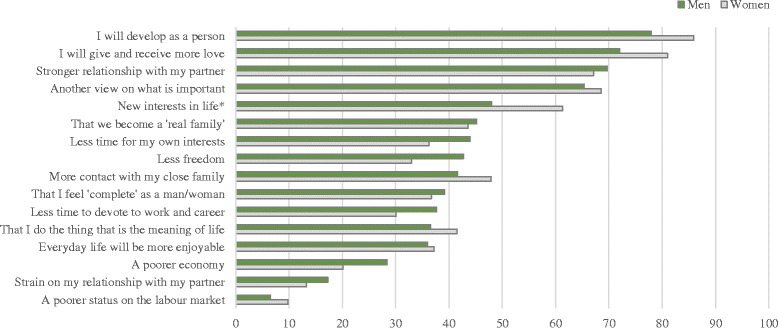
Perceived life changes. ªTo illustrate the participants’ main responses the total percentage for responses ‘important’ or ‘very important’ are presented. Significant effect of having children among women on variables: *p < 0.05

We used a questionnaire developed and pilot tested in a Swedish population and used in three studies on university students [22, 26, 27]. The instrument has shown good face validity and established internal consistency (Cronbach’s alpha >0.7) [22]. Modified versions of the questionnaire have also been used among university students in North America and Ukraine [25, 28]. The questionnaire comprises of 49 items that can be grouped into seven categories:

1. Socio-demographic characteristics and reproductive history (ten items): Age, bachelor degree program, type of housing, own and parents’ country of birth, relationship status, and own experience of pregnancy (yes/no).
2. Future intentions for childbirth (four items): *Do you wish to have children?* (yes/no). If the response was ‘yes’ three additional questions were posed: *How many children do you want? At what age do you want/did you have your first child? At what age do you want/did you have your last child?*
3. Importance of having children (one item): *How important is it for you to have children?* Response format was a visual analogue scale (VAS) (*unimportant* = 0 cm, *extremely important* = 10 cm).
4. Presumed behaviour in case of infertility (three items): *What would you do if you and your partner could not get pregnant?* Hereafter the participants were asked on a VAS to assess the likelihood of undergoing in vitro fertilization (IVF), adoption, or abstain from having children (*entirely unlikely* = 0 cm, *highly likely* = 10 cm).
5. Circumstances of importance for the decision to have children (13 items): Participant were asked to rate the importance of specific circumstances for their decision to become (or having become) a parent (see Fig. 1 for a comprehensive list). Response format was a Likert scale (1 *= unimportant,* 2 *= not very important,* 3 *= rather important,* 4 *= important,* 5 *= very important*) and the possibility of answering *no opinion*.
6. Positive and negative perceived life changes related to parenthood (nine items): Participants were asked to assess to what extent they agreed with items specifying possible (or experienced) life changes in relation to parenthood (see Fig. 2 for comprehensive list). Response format was a Likert scale (1 *= disagree,* 2 *= partially disagree,* 3 *= mainly agree,* 4 *= strongly agree,* 5 *= entirely*) and the possibility of answering *no opinion*.
7. Knowledge about fertility issues (nine items). This last category requested participants to answer questions about female age-related fertility, fecundity, and success odds for fertility treatment. The answer format was open-ended.

### Data analyses

Descriptive statistics included prevalences and means with standard deviations (SD), and medians with range (minimum – maximum). In order to compare the differences in answers between male and female students for continuous data, such as VAS scores, we used Mann–Whitney *U* test, as the data was not normally distributed. Pearson’s chi-squared test was used for nominal variables. Statistical significance was defined as a two-sided *P* value less than 0.05. All statistical analysis was performed using SPSS 22.0 software (IBM).

## Results

A total of 517 students answered the questionnaire, of these 79 men and 438 women. The socio-demographic characteristics and reproductive history are presented in Table 1. Mean age was 25.6 years among men and 24.2 years among women, and the majority of the participants were of Danish origin. Six out of ten reported to be in a steady relationship. A total of 9% of the men had children or were expecting children at the time of data collection. This was the case for 14% of the women (Table 1).

**Table 1:** Socio-demographic characteristics and reproductive history of all study participants (*N* = 517)^a^

	Men (*n* = 79)	Women (*n* = 438)
Age (years) mean (SD)	25.6 (4.4)	24.2 (5.1)
Range	21–47	18–58
Missing (n)	*0*	*1*
	n (%)	n (%)
Bachelor degree program		
Nursing	10 (13)	186 (42)
Midwifery	0	16 (4)
Physiotherapist	19 (24)	33 (7)
Occupational Therapy	6 (8)	26 (6)
Radiography	6 (8)	14 (3)
Social work	9 (11)	56 (13)
Biomedical Laboratory Sciences	6 (8)	20 (5)
Global Nutrition and Health	4 (5)	47 (11)
Emergency and Risk Management	6 (8)	0
Business and Public Administration	12 (15)	39 (9)
Missing	*1*	*1*
Housing type		
Student dormitory	9 (12)	45 (10)
Living with parents	6 (8)	46 (11)
Sublet Apartment	15 (19)	97 (22)
Lodging	15 (19)	66 (15)
Own apartment	33 (42)	180 (41)
Other	0	2 (1)
Missing	*1*	*2*
Ethnicity		
Born in Denmark	71 (90)	386 (88)
One/both parents born in Denmark	65 (82)	357 (82)
Missing	*0*	*2*
Steady relationship		
Yes	47 (60)	270 (62)
Missing data	*1*	*2*
Experienced pregnancy and outcome^a^		
Had children	6 (8)	47 (11)
Had abortion	10 (13)	39 (9)
Experienced miscarriage	2 (3)	11 (3)
Pregnant at present	1 (1)	13 (3)

^a^Percentages do not amount to 100, as the participant may have experienced more than one of the given outcomes

Table 2 shows that the majority of respondents, who did not have children at present (*n* = 453), wished this in the future (87% among men and 97% among women). This constituted a statistically significant difference between the genders (*p* = 0.002). The majority wanted to have two children. Three fourths reported that the most desired age to have a first child was 25–29 years. While no men wished to have children before this age, 8% of the women did so. With regards to the last child, the desired age for the majority of men was higher than that of the women, in that 55% of the men wanted to have their last child after the age of 34 years compared to 41% of the women, but overall there was no significant difference.

**Table 2:** Future intentions concerning having children^a^ (*n* = 453)

	Men		Women		*P*
	n	%	n	%	
Wish to have children					
Yes	59	87	354	97	0.002
No	9	13	12	3	
Missing	*4*		*15*		
Desired number					
1 or 1–2	2	4	23	7	0.37
2	26	46	126	37	
2–3	8	14	82	24	
3	14	25	84	24	
3–4 or more	6	11	28	8	
Missing	*16*		*38*		
Desired age at first child					
≤24 years	0	0	26	8	0.07
25–29 years	35	75	252	77	
30–34 years	10	21	42	13	
≥35 years	2	4	6	2	
Missing	*25*		*55*		
Desired age at last child					
≤29 years	1	3	34	12	0.21
30–34 years	15	42	134	47	
35–39 years	16	44	100	35	
>40 years	4	11	18	6	
Missing	*36*		*95*		

^a^Study participants with children or pregnant at present are not included

When asked about the importance of having children, women regarded this as being significantly more important than the men (*p* < 0.001) (Table 3). Women were more likely to believe that they would undergo IVF treatment in case of fertility problems (*p* < 0.001), while men were more likely to think that they would abstain from having children (*p* < 0.003). Adoption was considered an equally possible option by both (*p* = 0.2) (Table 3).

**Table 3:** Importance of children and presumed behaviour in case of infertility^a^

	Men			Women			*P*
	n	median	SD	n	median	SD	
Importance of children	77	8.2	3.1	428	9.7	2.3	<0.001
Missing	*2*			*10*			
Undergo IVF	74	6.4	3.4	427	8.9	2.7	<0.001
Missing	*5*			*11*			
Adoption	74	5.6	2.0	423	5.3	3.1	0.2
Missing	*5*			*15*			
Abstain from having children	73	2.0	2.7	421	0.4	2.5	0.003
Missing	*6*			*17*			

^a^Responses given on a visual analogue scale. For question on importance: 0 cm = unimportant and 10 cm = extremely important, and for behavioural questions: 0 cm = entirely unlikely and 10 m = highly likely

As seen in Fig. 1, the circumstances assessed as ‘important’ or ‘very important’ for the decision to become a parent by most participants were: Having a partner to share responsibility with, living in a stable relationship, and feeling sufficiently mature. Only approximately 1% listed friends planning/having children as an important issue. Significant differences between genders were seen for circumstances related to work and education, as more men than women rated having completed studies as important (*p* = 0.003). The same was seen for having started a career (and being in a permanent job). Not being ‘too old’ was regarded an important circumstance by 52% of men and 64% of women (*p* = 0.04) (Fig. 1).

When asked about perceived (or experienced) life changes in connection to becoming a parent, the most positively rated statement was personal development (78% men vs. 86% women) (Fig. 2). In addition, most participants stated that they ‘strongly’ or ‘entirely’ agreed that they would give and receive more love and that their relationship with partner would strengthen. While only 7% of men and 10% of women believed that having (or having had) children would negatively impact their status on the labor market, approximately one out of four thought it would affect their economy negatively. The only significant difference between men’s and women’s answers was related to the impact of parenthood on new interests in life (*p* = 0.04).

Table 4 shows that to questions on fertility knowledge answers were similar between the genders. Most participants answered correctly to women’s most fertile age period. Half of both genders thought a slight age-related decline in female fertility has its onset beyond the age of 30 years (correct answer: 25–29 years). More than 35% believed that a marked decrease does not occur until 40 years of age (correct answer: 35–39 years) - hereof more than 10% believed that this did not occur until 45 years. Both genders underestimated a couple’s ability to conceive if having unprotected sex at the time of ovulation. However, in comparison with women, men were significantly more likely to underestimate fecundity at ovulation, when the woman was aged 35–40 years (*p* = 0.047). For the question on how many couples experience involuntary infertility the majority answered incorrectly and overestimated the probability of achieving a child from IVF treatment, in that 40% of the men and 46% of the women indicated chances to be 40–100%.

**Table 4:** Knowledge about fertility issues^a^

Item	Categories	Men n (%)	Women n (%)	*P*
At what age are women the most fertile?	15–19 years	17 (23)	67 (15)	0.30
	20–24 years^a^	38 (50)	262 (61)	
	25–29 years	20 (26)	94 (22)	
	30–44 years	1 (1)	7 (2)	
	Missing	*3*	*8*	
At what age is there a slight decrease in women’s ability to become pregnant?	15–24 years	7 (9)	31 (7)	0.07
	25–29 years^a^	23 (30)	191 (45)	
	30–34 years	34 (45)	134 (31)	
	35–59 years	12 (16)	72 (17)	
	Missing	*3*	*10*	
At what age is there a marked decrease in women’s ability to become pregnant?	25–34 years	20 (26)	139 (33)	0.23
	35–39 years^a^	22 (29)	134 (31)	
	40–44 years	20 (26)	108 (25)	
	45–49 years	14 (19)	45 (11)	
	Missing	*3*	*12*	
If a young woman and man have unprotected intercourse at the time of ovulation - how large is the chance that she will become pregnant?	0–29%	18 (25)	61 (15)	0.06
	30–39%^a^	8 (11)	29 (7)	
	40–49%	4 (5)	17 (4)	
	50–100%	43 (59)	310 (74)	
	Missing	*6*	*21*	
If a woman and a man have regular unprotected intercourse during a period of 1 year:				
How large is the chance she will become pregnant if she is 25–30 years?	0–69%	35 (47)	182 (45)	0.06
	70–79%^a^	21 (29)	74 (18)	
	80–89%	9 (12)	98 (24)	
	90–100%	9 (12)	53 (13)	
	Missing	*5*	*31*	
How large is the chance she will become pregnant if she is 35–40 years?	0–49%	59 (80)	290 (72)	0.047
	50–59%^a^	8 (11)	57 (14)	
	60–69%	1 (1)	38 (10)	
	70–100%	6 (8)	17 (4)	
	Missing	*5*	*36*	
How many couples are involuntarily infertile?	0–4%	1 (1)	6 (2)	0.21
	5–9%	9 (12)	31 (8)	
	10–19%^a^	32 (43)	135 (34)	
	20–90%	33 (44)	226 (56)	
	Missing	*4*	*40*	
What are the chances on average of achieving a child from IVF treatment?	0–19%	12 (16)	60 (16)	0.82
	20–29%^a^	21 (29)	96 (25)	
	30–39%	11 (15)	52 (13)	
	40–100%	29 (40)	175 (46)	
	Missing	*6*	*55*	

^a^Categories marked with asterix contain the correct answer

## Discussion

In this study most of the participants, who did not have children, stated that they wished to have children in the future. Women regarded having children as more important than men did. If faced with infertility men were more likely to believe that they would abstain from having children and less likely to believe they would pursue IVF treatment. A clear majority stated that having a partner to share responsibility with was the most important circumstance for parenthood. However, significantly more men than women wanted to have completed their studies, to have started a career, and to be in a permanent job position, before having children. Significantly more women found it important to have children before being ‘too old’. Perceived (or experienced) life changes related to parenthood were overall positive, indicated by e.g. personal development, giving and receiving more love, and a stronger relationship with one’s partner. Knowledge about fertility issues was similar among both genders. Most respondents thought that both the slight and marked decrease in fertility occurs later than it really does, and at the same time more than half overestimated the ability of IVF treatment.

Strengths of this study include that we used a questionnaire originally developed and validated in a comparable Scandinavian population of Swedish university students [22, 26, 27]. All of the questions on fertility knowledge were open-ended to minimize the risk of an overestimation of knowledge. Another strength is the high participation rate of 99%. In addition, due to the sampling method, the random selection of respondents adds to the external validity. Unfortunately, those not present at the time of recruitment, could not be approached and, thus, no background variables are available for this group. In accordance with the 995 students registered for the module, teachers stated that attendance was approximately 50%. We do not know if non-participants potentially differ from students that attended lectures. In the present study only 15% (*n* = 79) of the participants were male students and care should be taken not to draw statistical conclusions. However, the percentage of men correlates well with the overall gender distribution at the inter-professional module being approximately 17% according to the Metropolitan University College Study Administration. Finally, most students at the given inter-professional module came from health related programmes, indicating that knowledge gaps regarding fertility may be more pronounced among the overall student population in Denmark.

Missing data always warrants special attention [29]. In the present study some of the questions could be perceived as personal and consequently be left unanswered. In an effort to limit this possible source of bias the questionnaire was anonymized, and this was stressed to participants beforehand. Still we encountered varying degrees of missing data for specific questions e.g. desired age for last child (25% for women and 50% missing data for men). Nevertheless, in many instances the answer was actually not left blank, but respondents simply answered with a question mark, thus indicating they had not reflected on the answers. Therefore, we believe that the missing answers may be attributed to non-reflection. Still we cannot know if this has led to an under- or overestimation of our results.

In line with previous studies the majority of our respondents wished to have children in the future. Further, our data showed a statistically significant gender difference on this question. This is in line with a recent study from the Ukraine [28]. Also, consistent with previous studies female students rated the importance of having a child and the likelihood of undergoing IVF treatment higher than did their male co-students [22, 25]. One bold interpretation of these findings is that women generally value having children higher than men, but at the same time there is accordance between the desired number of children and age of first child across genders. No gender differences emerged regarding parental age at birth of last child, but more than 60% of the women (significantly more than the men) agreed it is important to have children before being ‘too old’. A cautious interpretation of our results is that similarities and discrepancies between genders in their intentions for parenthood may interplay in the ‘negotiation’ about family planning within a couple and, thus, partly explain why parenthood is postponed.

In light of this interpretation Danish couples may benefit from tools like ‘My Reproductive Life Plan’ (RLP), recommended by the Centers for Disease Control and Prevention, and designed to help reflection on family planning matters [30, 31]. A Swedish randomized controlled trial study on RLP counseling found that the intervention strongly affected the age at which the participants wanted their last child [32]. The reflections encouraged by RLP may also refer to preconditions for parenthood e.g. work, studies, financial burden etc. In the present study the majority of high-scoring circumstances were related to creating the ‘ideal environment’ for having a child. Partner suitability was seen as highly important by both genders, which is mimicked by several other studies [22, 26, 28]. Still, men rated circumstances related to studies and work when making the decision to have children significantly higher than the women did. Another aspect is, that even though 86% of 30–39-year old fathers and 77% of mothers are in the labor market [19], only 50% of both men and women in our study reported access to childcare as an important or very important factor in the decision on having children. Explanations for this may be that parents are entitled to a combined parental leave of up to 52 weeks [20], and a total of 97% of all 3–5 year old children attend public daycare [19].

Previously published literature has compared men’s and women’s knowledge about fertility [22, 25, 26, 32, 33]. Some studies found that men overall had less knowledge about fertility [22, 25, 34], which is in line with the conclusion from Bunting et al., who investigated several knowledge areas including basics facts about infertility and indicators for reduced fertility [23]. In our study the participants generally lacked knowledge on fertility issues, but there were no substantial differences between the two genders.

The probability of pregnancy from unprotected intercourse in a young couple at the time of ovulation and the success rate of MAR were grossly overestimated by both groups [35]. Furthermore, more than half the participants answered incorrectly to female age at which a marked decrease in fertility is seen. Other studies have time and again found similar limited knowledge among university students [22, 25, 28, 36]. This is of particular concern, as a sizable percentage of respondents intend to have their last child at the age of 35 years or older, where a marked decline in female fertility is a reality [37]. The fact that many participants lacked knowledge on fertility raises concern and may indicate that both men and women are making the decision to postpone parenthood without being aware of possible consequences. Adding to this concern, the majority of participants stated wanting two children, but the current reproduction rate in Denmark is 1.69 [18] showing a potential disparity between what is desired and the actual outcome. The interaction between fertility knowledge and family intentions is not well documented, but a recent European study found that women, who under-estimated the impact of age on fertility, desired to have their first child at a higher age [34]. More studies are needed to confirm these results, but the necessity of fertility education on the ‘optimal’ timing of parenthood seems evident.

## Conclusion

The present study provides insight into contemporary attitudes towards parenthood and knowledge about fertility issues among Danish university college students. The majority of male and female respondents wished to have children and they had an overall positive attitude towards perceptions of parenthood. Many reported that they wished to have children at an age, where the biological decline in female fertility has set in, and there was a general lack of knowledge about fertility issues. Our findings indicate a need for additional information and counselling such as RLP to the younger populations in Denmark. RLP has shown to be useful to increase knowledge and awareness of reproductive health [30–32]. Our data did not indicate any strong associations between gender and knowledge about fertility. Future research should address the potential link between fertility knowledge and planning of parenthood, and we may benefit from intervention studies examining the effect of offering preconception care. Also, the authors suggest similar cross-sectional studies carried out in settings with larger proportions of male students and that does not have a focus on health related subjects.

## Abbreviations

IVF: In vitro fertilization
MAR: Medically assisted reproduction
RLP: ‘My Reproductive life-plan’
VAS: Visual analogue scale
